# Association of Extreme Heat With All-Cause Mortality in the Contiguous US, 2008-2017

**DOI:** 10.1001/jamanetworkopen.2022.12957

**Published:** 2022-05-19

**Authors:** Sameed Ahmed M. Khatana, Rachel M. Werner, Peter W. Groeneveld

**Affiliations:** 1 Division of Cardiovascular Medicine, Perelman School of Medicine, University of Pennsylvania, Philadelphia; 2 Penn Cardiovascular Outcomes, Quality, and Evaluative Research Center, Perelman School of Medicine, University of Pennsylvania, Philadelphia; 3 The Leonard Davis Institute of Health Economics, University of Pennsylvania, Philadelphia; 4 Division of General Internal Medicine, Perelman School of Medicine, University of Pennsylvania, Philadelphia; 5 Center for Health Equity Research and Promotion, Michael J. Crescenz Veterans Affairs Medical Center, Philadelphia, Pennsylvania

## Abstract

**Importance:**

The number of extreme heat events is increasing because of climate change. Previous studies showing an association between extreme heat and higher mortality rates generally have been limited to urban areas, and whether there is heterogeneity across different populations is not well studied; understanding whether this association varies across different communities, particularly minoritized racial and ethnic groups, may allow for more targeted mitigation efforts.

**Objective:**

To the assess the association between extreme heat and all-cause mortality rates in the US.

**Design, Setting, and Participants:**

This cross-sectional study involved a longitudinal analysis of the association between the number of extreme heat days in summer months from 2008 to 2017 (obtained from the Centers for Disease Control and Prevention’s Environmental Public Health Tracking Program) and county-level all-cause mortality rates (obtained from the National Center for Health Statistics), using a linear fixed-effects model across all counties in the contiguous US among adults aged 20 years and older. Data analysis was performed from September 2021 to March 2022.

**Exposures:**

The number of extreme heat days per month. Extreme heat was identified if the maximum heat index was greater than or equal to 90 °F (32.2 °C) and in the 99th percentile of the maximum heat index in the baseline period (1979 to 2007).

**Main Outcomes and Measures:**

County-level, age-adjusted, all-cause mortality rates.

**Results:**

There were 219 495 240 adults aged 20 years and older residing in the contiguous US in 2008, of whom 113 294 043 (51.6%) were female and 38 542 838 (17.6%) were older than 65 years. From 2008 to 2017, the median (IQR) number of extreme heat days during summer months in all 3108 counties in the contiguous US was 89 (61-122) days. After accounting for time-invariant confounding, secular time trends, and time-varying environmental and economic measures, each additional extreme heat day in a month was associated with 0.07 additional death per 100 000 adults (95% CI, 0.03-0.10 death per 100 000 adults; *P* = .001). In subgroup analyses, greater increases in mortality rates were found for older vs younger adults (0.19 death per 100 000 individuals; 95% CI, 0.04-0.34 death per 100 000 individuals), male vs female adults (0.12 death per 100 000 individuals; 95% CI, 0.05-0.18 death per 100 000 individuals), and non-Hispanic Black vs non-Hispanic White adults (0.11 death per 100 000 individuals; 95% CI, 0.02-0.20 death per 100 000 individuals).

**Conclusions and Relevance:**

These findings suggest that from 2008 to 2017, extreme heat was associated with higher all-cause mortality in the contiguous US, with a greater increase noted among older adults, men, and non-Hispanic Black individuals. Without mitigation, the projected increase in extreme heat due to climate change may widen health disparities between groups.

## Key Points

**Question:**

Is there an association between extreme heat and all-cause mortality in the US?

**Findings:**

In this cross-sectional study using a longitudinal analysis of county-level monthly all-cause mortality rates from all counties in the contiguous US from 2008 to 2017, each additional extreme heat day in a month was associated with 0.07 additional death per 100 000 adults.

**Meaning:**

These findings suggest that from 2008 to 2017 in the contiguous US, extreme heat was associated with higher adult all-cause mortality rates.

## Teaser

This cross-sectional study assesses the association between extreme heat and all-cause mortality rates in the US from 2008 to 2017.

## Introduction

Extreme heat events, or the occurrence of temperatures substantially hotter than typical, are increasing in intensity, frequency, and duration because of climate change.^1,2,3,4^ Such events will play an increasingly important role in health outcomes as the climate continues to warm. Understanding how extreme heat is associated with health outcomes in the US, particularly for vulnerable populations such as older adults and racially and ethnically underrepresented groups, is crucial for devising strategies to mitigate these outcomes.

The association between extreme heat and health outcomes, including mortality, has been noted previously.^5,6,7,8,9,10^ Although approximately 700 deaths per year are directly attributed to heat exposure in the US,^11^ the burden of excess deaths from any cause related to extreme heat is likely higher.^12^ Studies examining the association between extreme heat and mortality have been limited to certain parts of the US, typically urban areas, making the total burden of excess mortality related to extreme heat across the US unclear. Because the association between heat and human health is impacted by aspects of the built environment, such as tree cover and air conditioning use,^13,14^ understanding the burden of mortality due to extreme heat requires examining the potential association over a larger portion of the country, in both urban and nonurban areas.

Vulnerable and historically oppressed communities may be disproportionately affected by extreme heat because of differences in the burden of medical comorbidities, access to health care, and living in areas at greater risk of extreme heat exposure.^15^ Understanding which communities are disproportionately affected may allow for investments to guard against the adverse health outcomes associated with extreme heat. Although higher mortality among Black individuals explicitly indicated to be due to heat exposure has been noted,^11,16^ whether disparities exist when examining deaths from any cause, which is necessary to fully understand the extreme heat and mortality association, is unclear.

We hypothesize that extreme heat is associated with higher all-cause mortality across the US and that there is significant heterogeneity in this association between subgroups of age, sex, race, and ethnicity. To evaluate this hypothesis, we examined county-level all-cause mortality rates across the contiguous US in the years 2008 to 2017.

## Methods

This study was considered exempt according to the University of Pennsylvania institutional review board guidelines because it uses publicly available data; thus, informed consent was not sought in accordance with 45 CFR §46. This study follows the reporting requirements of the Strengthening the Reporting of Observational Studies in Epidemiology (STROBE) reporting guideline.^17^

### Extreme Heat

Extreme heat refers to temperatures that are substantially higher than typical for a given area.^18^ We used a combination of absolute and relative thresholds, as defined elsewhere,^7,19^ to determine the occurrence of extreme heat in every county in the contiguous US for each day in summer months (May to September) from 2008 to 2017. Heat index was used because this combines air temperature and relative humidity and indicates the temperature perceived by the human body.^20,21^ Daily maximum heat index levels in each county for summer months from 1979 to 2017 were obtained from the Centers for Disease Control and Prevention (CDC) Environmental Public Health Tracking Program.^19^ A baseline period of 1979 to 2007 was established, and the 99th percentile of the maximum heat index for each summer day during this period was calculated. An extreme heat day was defined as occurring when the maximum heat index was greater than or equal to 90 °F (32.2 °C) and in the 99th percentile of the maximum heat index in the baseline period for each county. The number of extreme heat days in each summer month from 2008 to 2017 was calculated. A heat wave was defined as 3 or more consecutive extreme heat days.

### Mortality

The number of deaths in each county from 2008 to 2017 was obtained from the National Center for Health Statistics. Data on each deceased person’s age, sex, race, ethnicity, cause of death, and month and year of death were also obtained. Mortality rates were age-adjusted by direct standardization to the 2000 US Census population.

Because rates in areas with small populations can be statistically unstable,^22^ we used spatial empirical Bayes smoothing, which stabilizes mortality rates by borrowing strength or confidence in the data from neighboring areas, with more populous areas contributing more to the smoothed estimate than less populous areas.^23^ Additional details on smoothing are in eAppendix 1 in the Supplement. Other data sources used are listed in eAppendix 2 in the Supplement.

### Outcomes

The primary outcome was the monthly, county-level, age-adjusted, empirical Bayes–smoothed, all-cause mortality rate for adults aged 20 years and older in summer months from 2008 to 2017. Secondary outcomes include mortality rates in subgroups based on age, sex, and race or ethnicity and stratified by county metropolitan status and the 2014 CDC Social Vulnerability Index (SVI) tertile, a measure of a community’s vulnerability to public health hazards, with higher values indicating a greater degree of vulnerability (eAppendix 3 in the Supplement).^24^ Deaths were classified by age, sex, and racial and ethnic group as indicated on death certificates, which have been shown to have a greater than 90% agreement with self-reported race and ethnicity for Black and White race and Hispanic ethnicity.^25^

### Statistical Analysis

We first calculated the total number of extreme heat days for each county during summer months from 2008 to 2017. Summary measures of county-level demographic, economic, structural, and environmental variables were calculated within quartiles of the total number of extreme heat days.

#### Fixed-Effects Model

To examine the association between the monthly number of extreme heat days and mortality rates, we fit a linear fixed-effects regression model. This longitudinal estimation technique controls for measured and unmeasured time-invariant confounding by examining the change in the dependent and independent variables for each subject (ie, county in this analysis).^26^ Month and year fixed effects accounted for secular time trends. Additional details are shown in eAppendix 4 in the Supplement. Time-varying, county-level demographic, economic, and health care–related measures included in the model are listed in eAppendix 5 in the Supplement. Demographic and economic covariates were chosen that potentially affect a community’s vulnerability to extreme heat according to the CDC’s National Environmental Public Health Tracking Network.^19^ In addition, comorbidities such as diabetes have been associated with heat-related mortality.^27^ Data for each covariate in the primary model were available for all counties across the entire study period. State-level cluster-robust SEs were used. The fixed-effects model was weighted by county population to account for differences in the underlying population sizes. Using the estimate for the association of mortality and extreme heat from the fixed-effects model, county population, and the number of extreme heat days, we estimated the number of additional deaths associated with extreme heat across the contiguous US.

#### Secondary and Sensitivity Analyses

In secondary analyses, we examined the association between extreme heat and all-cause mortality rates in the following subgroups: adults younger than 65 years, adults aged 65 years and older, men, women, Hispanic (any race) individuals, non-Hispanic Black individuals, non-Hispanic White individuals, and non-Hispanic other race (American Indian and Alaska Native, Asian, Native Hawaiian and other Pacific Islander, or identifying with 2 or more races) individuals. We also performed a stratified analysis based on the county metropolitan status and tertiles of county CDC SVI values. To assess whether the association differed between subgroups, we fit fixed-effects models that simultaneously estimated regression coefficients for the subgroups of interest and also included interaction terms between the subgroup indicator and the independent variables.^28^

In sensitivity analyses, we refit the primary fixed-effects models with additional variables that were not available for all counties or in all years of the study period, including the monthly number of days with air quality that was unhealthy for sensitive groups or worse (available in 1019 to 1027 counties over the study period) and the percentage of county land with forest cover and the percentage of county land developed (available for all counties for the years 2008, 2011, 2013, and 2016) because these factors may confound the association between extreme heat and mortality.^13,29^ To assess whether the findings of our analysis were sensitive to the definition of extreme heat, we used alternative definitions for extreme heat (≥90 °F [32.2 °C] and in the 95th or 90th percentile of the maximum heat index in the baseline period, respectively). We also evaluated the association between occurrence of heat waves and mortality.

Summary measures are presented as mean (SD or 95% CI) or median (IQR). Two-sided *P* ≤ .05 was considered significant. Empirical Bayes smoothing was performed using GeoDa open source statistical software version 1.14.0 (University of Chicago). All other statistical analyses were conducted using Stata statistical software version 17 (StataCorp). Data analysis was performed from September 2021 to March 2022.

## Results

**Figure 1.**
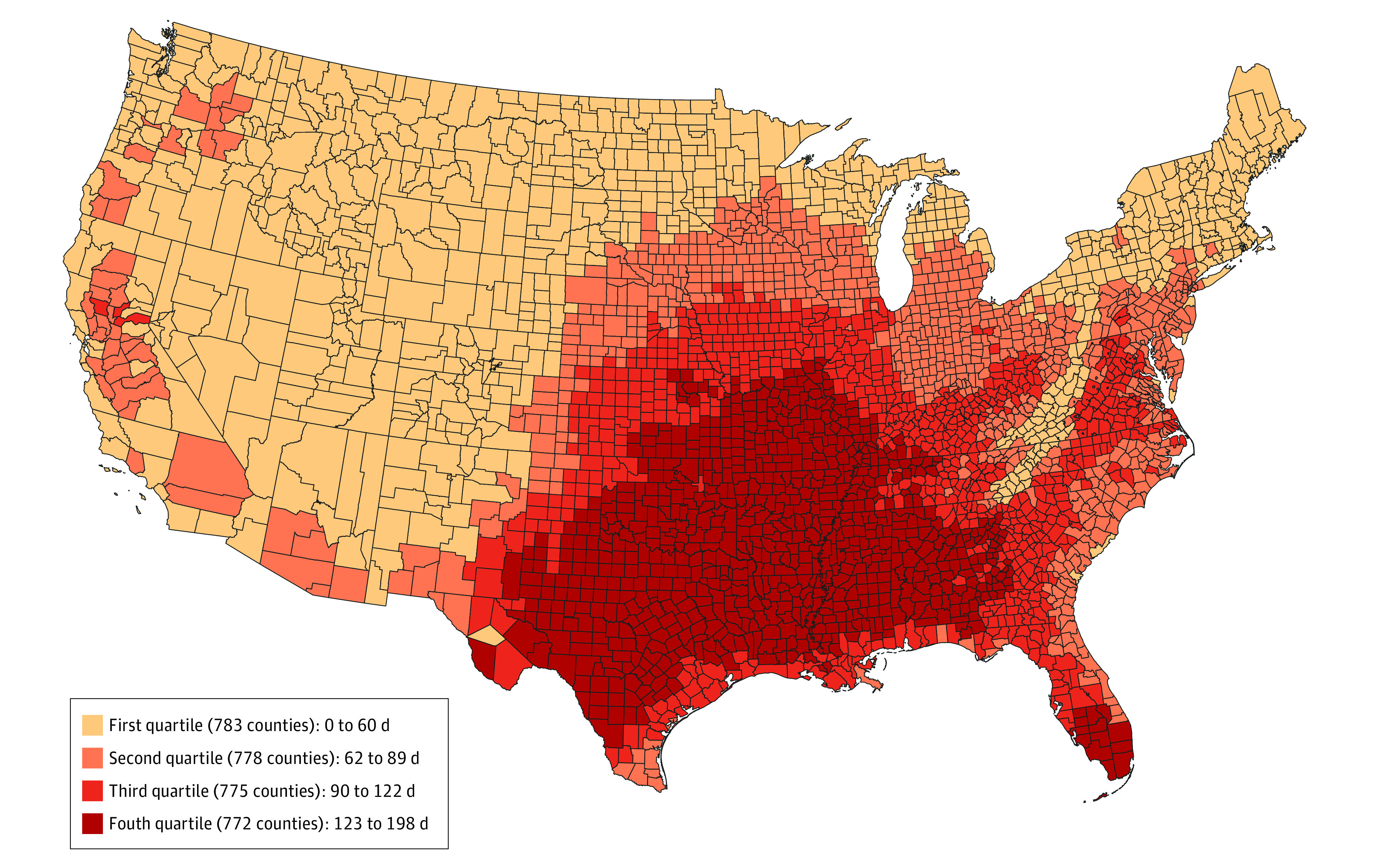
Total Number of Extreme Heat Days in Summer Months (May to September) From 2008 to 2017 An extreme heat day was designated if the maximum heat index on that day was greater than or equal to 90 °F (32.2 °C) and in the 99th percentile of the maximum heat index in the baseline period (1979 to 2007).

All 3108 counties in the contiguous US were included. There were 219 495 240 adults aged 20 years and older residing in these counties in 2008, of whom 113 294 043 (51.6%) were female and 38 542 838 (17.6%) were older than 65 years. The median (IQR) 10-year total number of extreme heat days during summer months from 2008 to 2017 was 89 (61-122) days. There were 0 to 61 extreme heat days in the first quartile, 62 to 89 in the second quartile, 90 to 122 in the third quartile, and 123 to 198 in the fourth quartile of counties (Figure 1). The median (IQR) county adult population (in 2008) was 17 387 (5668-46 058) individuals in the first quartile, 29 566 (12 018-86 415) individuals in the second quartile, 16 720 (8009-39 975) individuals in the third quartile, and 16 810 (8814-34 392) individuals in the fourth quartile (Table 1). The median (IQR) proportion of non-Hispanic Black residents was 0.4% (0.2%-1.2%) in the first quartile, 1.7% (0.4%-8.3%) in the second quartile, 3.8% (0.6%-14.5%) in the third quartile, and 7.0% (1.5%-22.6%) in the fourth quartile. The median (IQR) poverty rate was 12.9% (10.4%-15.9%) in the first quartile, 12.4% (9.8%-16.4%) in the second quartile, 14.7% (11.2%-18.8%) in the third quartile, and 17.6% (14.4%-21.55%) in the fourth quartile. Other county-level economic, demographic, and health care–related variables for each quartile are listed in Table 1.

**Table 1.** County Characteristics by Quartile of Total Number of Extreme Heat Days^a^

Characteristic	Median (IQR)			
	First quartile (n = 783)	Second quartile (n = 778)	Third quartile (n = 775)	Fourth quartile (n = 772)
Total extreme heat days across study period, No.	34 (7-48)	78 (72-84)	101 (95-113)	141 (130-153)
Population (≥20 y), No.	17 387 (5668-46 058)	29 566 (12 018-86 415)	16 720 (8009-39 975)	16 810 (8814-34 392)
County adult residents, %				
Aged ≥65 y	21.6 (18.3-25.6)	20.2 (17.5-23.2)	20.4 (17.7-23.4)	20.9 (18.0-23.7)
Female	49.5 (48.7-50.5)	48.7 (48.0-49.5)	48.6 (47.9-49.4)	48.5 (47.6-49.5)
Male	49.5 (48.7-50.5)	48.7 (48.0-49.5)	48.6 (47.9-49.4)	48.5 (47.6-49.5)
Hispanic (any race)	2.4 (1.5-4.7)	1.6 (1.0-3.2)	1.4 (1.1-2.2)	1.6 (1.1-2.5)
Non-Hispanic				
Black	0.4 (0.2-1.2)	1.7 (0.4-8.3)	3.8 (0.6-14.5)	7.0 (1.5-22.6)
White	93.0 (84.5-96.3)	91.4 (75.6-96.5)	88.5 (70.9-96.0)	77.2 (63.5-89.3)
Other race^b^	2.2 (1.0-6.6)	2.5 (1.1-5.9)	2.4 (1.2-5.1)	2.5 (1.3-8.6)
Unemployment rate	4.7 (3.5-5.9)	5.1 (4-6.2)	5.2 (3.8-6.3)	5.1 (4.1-6.5)
Poverty rate	12.9 (10.4-15.9)	12.4 (9.8-16.4)	14.7 (11.2-18.8)	17.6 (14.4-21.55)
Adults (aged 18-64 y) without health insurance, %	18.6 (14.8-23.6)	17.1 (13.9-21.6)	20.1 (16.3-24.4)	25.0 (20.5-28.6)
Adults with diabetes, %	7.3 (6.5-8.2)	8.6 (7.3-9.9)	9.5 (8.1-10.9)	9.7 (8.6-11.2)
County land forested, %	33.6 (5.5-58.0)	16.3 (4.0-38.8)	26.5 (6.1-51.9)	29.5 (9.8-49.3)
County land developed, %^c^	3.7 (1.6-7.3)	7.7 (5.2-12.9)	6.6 (4.9-10.2)	5.3 (4.1-7.3)
Household income, $	43 467 (38 439-50 370)	45 437 (39 754-52 152)	42 206 (36 815-48 039)	37 696 (33 116-43 387)
Primary care practitioners per 100 000 adult residents, No.	81.9 (49.0-116.8)	72.4 (46.9-105.3)	63.9 (36.3-92.5)	54.0 (31.8-81.6)
Hospital beds per 100 000 adult residents, No.	324.0 (141.1-612.6)	292.1 (130.3-505.2)	330.2 (88.3-613.8)	313.6 (118.2-555.0)
Total poor air quality days across study period, No.^d^	14 (3-37)	25 (4-64)	18 (3-37)	16 (1-43)
Social Vulnerability Index, No.^e^	0.35 (0.17-0.57)	0.41 (0.19-0.67)	0.54 (0.26-0.77)	0.71 (0.49-0.86)
Metropolitan counties, No. (%)^f^	219 (28.0)	369 (47.4)	317 (40.9)	255 (33.0)
US Census regions, No. (%)				
Midwest	227 (29.0)	401 (51.5)	276 (35.6)	151 (19.6)
Northeast	144 (18.4)	71 (9.1)	2 (0.3)	0
South	64 (8.2)	248 (31.9)	490 (63.2)	620 (80.3)
West	348 (44.4)	58 (7.5)	7 (0.9)	1 (0.1)

^a^ An extreme heat day was designated if the maximum heat index on that day was greater than or equal to 90 °F (32.2 °C) and in the 99th percentile of the maximum heat index in the baseline period (1979 to 2007). All covariates are from the baseline year (2008) unless specified otherwise.

^b^ Non-Hispanic other race population consists of county-level estimates of individuals identified as non-Hispanic and American Indian and Alaska Native, Asian, Native Hawaiian and other Pacific Islander, or identifying with 2 or more races according to the US Census Bureau.

^c^ Includes low, medium, and high intensity development based on the Anderson Land Cover Classification System.

^d^ Refers to the monthly number of days with air quality that was unhealthy for sensitive groups or worse (available in 1019 to 1027 counties over the study period). Air Quality Index values were obtained from the Environmental Protection Agency.

^e^ Based on the 2014 Centers for Disease Control and Prevention Social Vulnerability Index (eAppendix 3 in the Supplement). Higher values indicate a greater degree of vulnerability to public health hazards.

^f^ Based on the 2013 National Center for Health Statistics Urban-Rural Scheme.

### Primary Analysis

**Figure 2.**
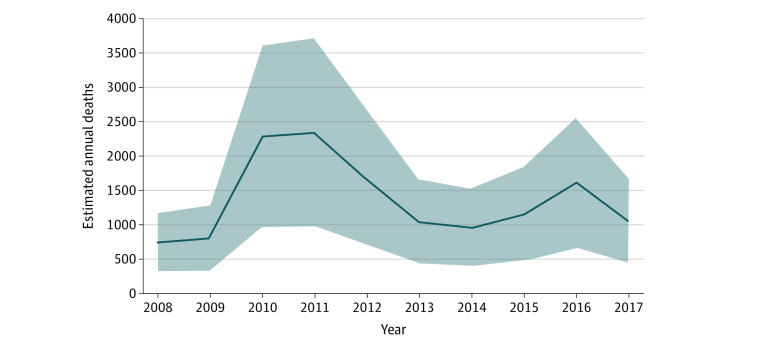
Annual Estimated Additional Deaths Associated With Extreme Heat Days During Summer Months An extreme heat day was designated if the maximum heat index on that day was greater than or equal to 90 °F (32.2 °C) and in the 99th percentile of the maximum heat index in the baseline period (1979 to 2007). Shaded areas represent 95% CIs of annual estimates.

In the primary fixed-effects model, each additional extreme heat day in a month was associated with 0.07 additional death per 100 000 individuals (95% CI, 0.03-0.10 additional death per 100 000 individuals; *P* = .001) among adults aged 20 years or older (Table 2). On the basis of the annual population and monthly number of extreme heat days in each county, extreme heat days were associated with an estimated mean of 1373 additional deaths per year (95% CI, 584-2163 additional deaths per year) among adults during summer months from 2008 to 2017 in the contiguous US (Figure 2 and eTable 1 in the Supplement). Estimated annual excess deaths varied from 752 (95% CI, 319-1184) in 2008 to 2337 (95% CI, 993-3681) in 2011.

**Table 2.** Linear Fixed-Effects Regression Model Outcome: County-Level, Monthly Age-Adjusted All-Cause Mortality Rate for Adults (≥20 Years)

Population	Additional deaths per 100 000 individuals associated with 1 additional extreme heat day per month^a^		Annual estimated deaths associated with extreme heat days from 2008 to 2017, mean (95% CI)^b^
	Estimate (95% CI)	*P* value	
Primary analysis, all adults (aged ≥20 y)	0.07 (0.03 to 0.12)	.001	1373 (584 to 2163)
Secondary analyses			
Age 20-64 y	0.04 (0.02 to 0.06)	.001	562 (234 to 890)
Age ≥65 y	0.23 (0.07 to 0.38)	.01	802 (247 to 1358)
Female	0.02 (−0.02 to 0.06)	.25	225 (−165 to 615)
Male	0.14 (0.08 to 0.20)	<.001	1262 (709 to 1815)
Hispanic (any race)	0.08 (0.002 to 0.16)	.045	219 (5 to 432)
Non-Hispanic			
Black	0.18 (0.10 to 0.25)	<.001	464 (269 to 660)
White	0.07 (0.01 to 0.12)	.02	801 (119 to 1483)
Other race^c^	0.07 (−0.04 to 0.18)	.23	76 (−49 to 200)
Counties (all adults)^d^			
Metropolitan	0.09 (0.04 to 0.13)	<.001	1342 (640 to 2043)
Nonmetropolitan	0.03 (−0.03 to 0.09)	.34	83 (−90 to 256)
Social Vulnerability Index, tertile^e^			
First	0.10 (0.04 to 0.16)	.001	449 (188 to 710)
Second	0.07 (0.02 to 0.11)	.01	481 (152 to 809)
Third	0.05 (−0.001 to 0.11)	.05	378 (−7 to 763)
Alternative extreme heat definitions			
≥90 °F (32.2 °C) and in the 95th percentile of the maximum heat index in the baseline period (all adults)	0.06 (0.03 to 0.08)	<.001	1839 (991 to 2687)
≥90 °F (32.2 °C) and in the 90th percentile of the maximum heat index in the baseline period (all adults)	0.05 (0.02 to 0.07)	<.001	1992 (957 to 3027)

^a^ An extreme heat day was designated if the maximum heat index on that day was greater than or equal to 90 °F (32.2 °C) and in the 99th percentile of the maximum heat index in the baseline period (1979 to 2007).

^b^ Estimates are based on annual adult population and then number of extreme heat days in each county from 2008 to 2017.

^c^ Non-Hispanic other race population consists of county-level estimates of individuals identified as non-Hispanic and American Indian and Alaska Native, Asian, Native Hawaiian and other Pacific Islander, or identifying with 2 or more races according to the US Census Bureau.

^d^ Based on the 2013 National Center for Health Statistics Urban-Rural Scheme.

^e^ Based on the 2014 Centers for Disease Control and Prevention Social Vulnerability Index (eAppendix 3 in the Supplement). Higher values indicate a greater degree of vulnerability to public health hazards.

### Secondary Analyses

In subgroup analyses, each additional extreme heat day per month was associated with a significantly higher monthly mortality rate for both adults aged 20 to 64 years (0.04 death per 100 000 individuals; 95% CI, 0.02 to 0.06 death per 100 000 individuals) and those older than 64 years (0.23 death per 100 000 individuals; 95% CI, 0.07 to 0.38 death per 100 000 individuals) (Table 2). The magnitude of the association was higher among adults older than 64 years compared with younger adults (0.19 additional death per 100 000 individuals per month; 95% CI, 0.04 to 0.34 additional death per 100 000 individuals per month) (Table 3). The association was significant for men but not for women, and the male mortality rates were higher than female mortality rates (0.12 death per 100 000 individuals per month; 95% CI, 0.05 to 0.18 death per 100 000 individuals per month). Among racial and ethnic groups, the association was significant for non-Hispanic Black individuals (0.18 death per 100 000 individuals; 95% CI, 0.07 to 0.23 death per 100 000 individuals), non-Hispanic White individuals (0.07 death per 100 000 individuals; 95% CI, 0.01 to 0.12 death per 100 000 individuals), and Hispanic (any race) individuals (0.08 death per 100 000 individuals; 95% CI 0.002 to 0.16 death per 100 000 individuals), but not non-Hispanic other race individuals (0.07 death per 100 000 individuals; 95% CI, −0.04 to 0.18 death per 100 000 individuals) (Table 2). The monthly mortality rate was higher among non-Hispanic Black individuals than among non-Hispanic White individuals (0.11 death per 100 000 individuals; 95% CI, 0.02 to 0.20 death per 100 000 individuals) (Table 3). The mortality rates did not differ significantly between non-Hispanic White individuals and Hispanic (any race) or non-Hispanic other race individuals. The association between extreme heat and all-cause mortality was significant in metropolitan counties (0.09 death per 100 000 individuals; 95% CI, 0.04 to 0.13 death per 100 000 individuals) but not nonmetropolitan counties (0.03 death per 100 000 individuals; 95% CI, −0.03 to 0.09 death per 100 000 individuals) (Table 2). The association was significant among counties in the least socially vulnerable counties according to tertiles of the SVI (first tertile, 0.10 death per 100 000 individuals; 95% CI, 0.04 to 0.16 death per 100 000 individuals; second tertile, 0.07 death per 100 000 individuals; 95% CI, 0.02 to 0.11 death per 100 000 individuals), but not in the most socially vulnerable counties (third tertile, 0.05 death per 100 000 individuals; 95% CI, −0.001 to 0.11 death per 100 000 individuals). The associations for stratifications of metropolitan status and SVI tertiles did not differ significantly from each other (Table 3).

**Table 3.** Comparison of Association Between the Number of Extreme Heat Days per Month and Monthly All-Cause Mortality Rates Between Subgroups^a^

Subgroups	Additional deaths per 100 000 individuals associated with 1 additional extreme heat day per month vs reference group, estimate (95% CI)^b^	*P* value
Age, y		
20-64	1 [Reference]	.01
≥65	0.19 (0.04 to 0.34)	
Sex		
Female	1 [Reference]	<.001
Male	0.12 (0.05 to 0.18)	
Race and ethnicity		
Hispanic (any race)	0.02 (−0.09 to 0.13)	.75
Non-Hispanic		
Black	0.11 (0.02 to 0.20)	.02
White	1 [Reference]	NA
Other race^c^	0.003 (−0.12 to 0.12)	.96
County metropolitan status (all adults)^d^		
Metropolitan	0.06 (−0.004 to 0.12)	.07
Nonmetropolitan	1 [Reference]	
County Social Vulnerability Index, tertile^e^		
First	1 [Reference]	NA
Second	−0.04 (−0.09 to 0.02)	.19
Third	−0.05 (−0.12 to 0.02)	.14

Abbreviation: NA, not applicable.

^a^ An extreme heat day was designated if the maximum heat index on that day was greater than or equal to 90 °F (32.2 °C) and in the 99th percentile of the maximum heat index in the baseline period (1979 to 2007).

^b^ Regression coefficient is the interaction between indicator for subgroup and the number of extreme heat days per month.

^c^ Non-Hispanic other race population consists of county-level estimates of individuals identified as non-Hispanic and American Indian and Alaska Native, Asian, Native Hawaiian and other Pacific Islander, or identifying with 2 or more races according to the US Census Bureau.

^d^ Based on the 2013 National Center for Health Statistics Urban-Rural Scheme.

^e^ Based on the 2014 Centers for Disease Control and Prevention Social Vulnerability Index (eAppendix 3 in the Supplement). Higher values indicate a greater degree of vulnerability to public health hazards.

The association between extreme heat days and all-cause mortality was essentially unchanged when the number of poor air quality days per month or the proportion of land that is forested (0.08 death per 100 000 individuals; 95% CI, 0.03-0.12 death per 100 000 individuals) and land that is developed (0.08 death per 100 000 individuals; 95% CI, 0.05-0.16 death per 100 000 individuals) were included in the primary model. Using the 2 alternative definitions of extreme heat days (in the 95th and 90th percentile of the maximum heat index in baseline period, respectively), extreme heat was associated with 0.06 additional death per 100 000 individuals per month (95% CI, 0.03-0.08 additional death per 100 000 individuals per month) and 0.05 additional death per 100 000 individuals per month (95% CI, 0.02-0.07 additional death per 100 000 individuals per month), respectively, with an estimated mean annual excess deaths of 1838 (95% CI, 991-2687 deaths) and 1992 (95% CI, 957-3027 deaths), respectively (Table 2 and eTable 2 and eTable 3 in the Supplement). The occurrence of a heat wave was associated with 0.27 additional death per 100 000 individuals per month (95% CI, 0.01-0.53 death per 100 000 individuals per month).

## Discussion

In this cross-sectional study, by use of a longitudinal analysis we found that in the contiguous US from 2008 to 2017, extreme heat days were associated with higher county-level monthly adult all-cause mortality rates. There was significant heterogeneity in the magnitude of the association among subgroups based on age, sex, race, and ethnicity. The association between extreme heat and all-cause mortality was significantly greater for non-Hispanic Black compared with non-Hispanic White adults, for men compared with women, and for older compared with younger adults.

The association between extreme heat and higher mortality rates has been noted previously.^5,8,30,31^ Extreme heat causes morbidity and mortality through a wide variety of mechanisms and by impacting most organ systems.^32,33^ Individuals with impaired thermoregulation, such as the elderly and those with medical comorbidities, are particularly vulnerable to the health effects of extreme heat. Most previous studies in the US have examined this association only in large urban areas and, thus, provide incomplete understanding of the excess mortality associated with extreme heat across the country. In our analysis, which included all counties in the contiguous US, we found that extreme heat days were associated with a mean of 1373 (95% CI, 584-2163) to 1992 (95% CI, 957-3027) additional deaths per year, depending on the definition of extreme heat used. This estimate is higher than the approximately 700 annual deaths directly ascribed to heat exposure during this same period.^11^ Although differences in methods make comparison with estimates from other studies challenging, 1 analysis^5^ of 297 US counties (representing approximately 60% of the population) estimated a mean of approximately 2300 additional deaths per year associated with extreme heat from 1997 to 2006. The estimated number of additional deaths per year in our analysis varied from year to year, with the highest number occurring from 2010 to 2012, likely related to the occurrence of several extreme heat events during those years.^34,35,36^ With the projected continued increase in the number of extremely hot days in the US resulting in 20 to 30 additional extreme heat days per year in most areas across the US by the middle of the 21st century,^37^ without adaptations, the number of deaths associated with extreme heat may increase further.

The association of extreme heat and mortality was heterogenous across population subgroups based on age, sex, and race and ethnicity. A higher mortality rate in minoritized groups when examining heat-specific mortality has been noted previously.^11,16^ In our analysis, we found that the association between extreme heat and all-cause mortality was significantly greater for non-Hispanic Black compared with non-Hispanic White adults. Potential reasons may include a greater proportion of non-Hispanic Black individuals residing in urban areas, which may be more prone to the effects of extreme heat because of various factors, such as the heat island effect (ie, the phenomenon of urban areas experiencing higher temperatures than surrounding areas because of the presence of structures such as buildings and roads, which absorb heat more than natural terrain).^38,39^ Although we did not find significant differences in the association between extreme heat and mortality for all adults between counties with different levels of social vulnerability to public health hazards, such county-level measures likely do not fully account for the substantial differences in socioeconomic factors and lower rates of access to quality health care by ethnically and racially underrepresented groups in the US, which may play a role in the differences noted. This finding suggests that, like many other public health challenges in the US, extreme heat has had a disproportionately higher impact on the health of minoritized groups such as non-Hispanic Black individuals. This disparity is highlighted even more by a recent study^40^ suggesting lower per capita carbon emissions among Black compared with White households. The projected continued increase in extreme heat, therefore, may be associated with a widening of preexisting health disparities in the US. These results also suggest the possibility that interventions to improve the resilience of communities that are particularly at risk to extreme heat through interventions such as increasing tree cover may play a role in narrowing such disparities.^13^

### Limitations

Our study has certain limitations. As an observational study, the associations noted cannot be concluded as being causal. Because all data were aggregated at the county level, inferences at the individual level are not possible. Although the fixed-effects regression model accounted for both measured and unmeasured time-invariant confounding and secular time trends, other sources of bias, such as residual time-varying confounding, are possible. However, we attempted to minimize the impact of unmeasured time-varying confounding by including several important potential confounders. In the subgroup analyses, the county-level socioeconomic and health care–related covariates used were for the entire county population, and not just for the subgroup being analyzed, which may be a source of residual confounding.

## Conclusions

Extreme heat days in the contiguous US from 2008 to 2017 were associated with higher county-level, all-cause mortality rates among adults. There were significant differences in the association between different subgroups, with a higher mortality rates noted for older adults, men, and non-Hispanic Black adults. With extreme heat projected to increase substantially over the coming decades, vulnerable populations in the US are likely to bear a disproportionate burden of its adverse health effects.

## Notes

Supplement. **eAppendix 1.** Spatial Empirical Bayes Smoothing **eAppendix 2.** Other Data Sources **eAppendix 3.** CDC Social Vulnerability Index **eAppendix 4.** Fixed-Effects Model **eAppendix 5.** Fixed-Effects Model Covariates **eTable 1.** Annual Estimated Additional Deaths Associated With Extreme Heat Days During Summer Months, With Extreme Heat Defined as ≥90°F (32.2°C) and in the 99th Percentile of the Maximum Heat Index in the Baseline Period (1979-2007) **eTable 2.** Annual Estimated Additional Deaths Associated With Extreme Heat Days During Summer Months, With Extreme Heat Defined as ≥90°F (32.2°C) and in the 95th Percentile of the Maximum Heat Index in the Baseline Period (1979-2007) **eTable 3.** Annual Estimated Additional Deaths Associated With Extreme Heat Days During Summer Months, With Extreme Heat Defined as ≥90°F (32.2°C) and in the 90th Percentile of the Maximum Heat Index in the Baseline Period (1979-2007)
eReferences
