# Supplementary material for: Association of Extreme Heat With All-Cause Mortality in the Contiguous US, 2008-2017
Source: JAMA Netw Open. 2022 May 19;5(5):e2212957. doi: 10.1001/jamanetworkopen.2022.12957 (PMC9121188; doi:10.1001/jamanetworkopen.2022.12957)
Supplement: Supplement. — eAppendix 1. Spatial Empirical Bayes Smoothing eAppendix 2. Other Data Sources eAppendix 3. CDC Social Vulnerability Index eAppendix 4. Fixed-Effects Model eAppendix 5. Fixed-Effects Model Covariates eTable 1. Annual Estimated Additional Deaths Associated With Extreme Heat Days During Summer Months, With Extreme Heat Defined as ≥90°F (32.2°C) and in the 99th Percentile of the Maximum Heat Index in the Baseline Period (1979-2007) eTable 2. Annual Estimated Additional Deaths Associated With Extreme Heat Days During Summer Months, With Extreme Heat Defined as ≥90°F (32.2°C) and in the 95th Percentile of the Maximum Heat Index in the Baseline Period (1979-2007) eTable 3. Annual Estimated Additional Deaths Associated With Extreme Heat Days During Summer Months, With Extreme Heat Defined as ≥90°F (32.2°C) and in the 90th Percentile of the Maximum Heat Index in the Baseline Period (1979-2007) eReferences [file jamanetwopen-e2212957-s001.pdf]

Supplemental Online Content

Khatana SAM, Werner RM, Groeneveld PW. Association of extreme heat with all-cause mortality in the contiguous US, 2008-2017. *JAMA Netw Open*. 2022;5(5):e2212957. doi:10.1001/jamanetworkopen.2022.12957

eAppendix 1. Spatial Empirical Bayes Smoothing

eAppendix 2. Other Data Sources

eAppendix 3. CDC Social Vulnerability Index

eAppendix 4. Fixed-Effects Model

eAppendix 5. Fixed-Effects Model Covariates

eTable 1. Annual Estimated Additional Deaths Associated With Extreme Heat Days During Summer Months, With Extreme Heat Defined as ≥90°F (32.2°C) and in the 99th Percentile of the Maximum Heat Index in the Baseline Period (1979-2007)

eTable 2. Annual Estimated Additional Deaths Associated With Extreme Heat Days During Summer Months, With Extreme Heat Defined as ≥90°F (32.2°C) and in the 95th Percentile of the Maximum Heat Index in the Baseline Period (1979-2007)

eTable 3. Annual Estimated Additional Deaths Associated With Extreme Heat Days During Summer Months, With Extreme Heat Defined as ≥90°F (32.2°C) and in the 90th Percentile of the Maximum Heat Index in the Baseline Period (1979-2007)

eReferences

This supplemental material has been provided by the authors to give readers additional information about their work.

© 2022 Khatana SAM et al. *JAMA Network Open.*

eAppendix 1: Spatial Empirical Bayes Smoothing

Mortality rate estimates from areas with small populations are prone to instability with potentially large changes seen in the mortality rate due to a small change in the absolute number of deaths. This may not provide an accurate assessment of the risk of mortality in these areas. To account for this instability, we used spatial empirical Bayes smoothing of the mortality rates. Empirical Bayes smoothing combines the raw mortality rate with a reference mortality rate and calculates a weighted average of the two with weights that are directly proportional to the population at risk. Therefore, counties with small populations will have their rates adjusted to a greater degree than counties with a larger population. As in other Bayesian frameworks, a prior distribution is specified, and after observing data, a posterior distribution is obtained.

The standard approach for Bayesian estimation of rates is to specify a Poisson distribution for the observed counts (deaths in this case) and a Gamma distribution prior. In an Empirical Bayes approach, parameters for the prior Gamma distribution are estimated from the actual data. The estimated prior rate can be considered the reference rate.

The empirical Bayes smoothed rate for a given county *i* is estimated using the following equation:

$$\text{Smoothed Rate}_i = \omega_i \times \text{crude rate}_i + (1-\omega_i) \times \text{reference rate}_i$$

where ω is a weight parameter calculated as follows:

$$\omega_i = \frac{\sigma^2}{(\sigma^2 + \mu/Population_i)}$$

where σ^2 and μ represent the variance and mean of the prior distribution and $Population_i$ refers to the population of county *i*.

μ is the reference mortality rate and is calculated as follows:

© 2022 Khatana SAM et al. *JAMA Network Open.*

$$\sum_{i=1}^{i=n} Observed\ Deaths_i \,/ \sum_{i=1}^{i=n} Population_i$$

and the σ^2 as follows:

$$\frac{\sum_{i=1}^{1=n} Population_i (crude\ rate_i - \mu)^2}{\sum_{i=1}^{i=n} Population_i} - \frac{\mu}{\sum_{i=1}^{i=n} Population_i / n}$$

where n refers to the number of counties in the reference sample.

In spatial empirical Bayes, the mean and variance of the prior are estimated from a localized group of observations rather than the global sample (i.e. all counties in the US). In our analysis, we used all neighboring counties as the reference group for each county.

© 2022 Khatana SAM et al. *JAMA Network Open.*

eAppendix 2: Other Data Sources

Sources for other county-level variables were as follows: monthly mean precipitation levels (in inches) from the Centers for Disease Control and Prevention Environmental Public Health Tracking Program, monthly percentage of the county population living in areas with moderate or higher drought from the US Drought Monitor, monthly number of disaster declarations from the Federal Emergency Management Agency, monthly unemployment rate by the Bureau for Labor Services, daily Air Quality Index levels from the Environmental Protection Agency, total population, proportion of residents in different sub-groups based on age, gender, race and ethnicity, percentage of residents living in poverty, median household income, percentage of 18 to 64 year old adults without health insurance, and county metropolitan status (in 2010) from the U.S. Census Bureau. The percentage of county land covered by forest and the percentage of land developed (low, median and high intensity development) were obtained from the Multi-Resolution Land Characteristics Consortium National Land Cover Database.

© 2022 Khatana SAM et al. *JAMA Network Open.*

eAppendix 3: CDC Social Vulnerability Index

The CDC Social Vulnerability Index (SVI) is a measure of an area's vulnerability to public health hazards.[1] The SVI has been associated with health outcomes in previous studies.[2-4] The SVI consists of 15 variables grouped into 4 categories: 1) socioeconomic, 2) household composition and disability, 3) minority status and language, and 4) housing and transportation. Each area is ranked on a scale of 0 to 1 on each factor, and the SVI is the unweighted mean of these ranks. A higher value indicates greater vulnerability. All variables are derived from US Census Bureau estimates. The 2014 version of the SVI was used for this analysis.

Components of the Social Vulnerability Index

**Socioeconomic Status Index**
Proportion of residents with income below poverty level
Proportion of residents unemployed
Median household income
Proportion of residents without high school diploma
**Household Composition and Disability Index**
Proportion of residents who are 65 years of age or older
Proportion of residents who are 17 years of age or younger
Proportion of civilian residents with a disability
Proportion of households that are single-parent households
**Minority Status and Language Index**
Proportion of residents who are not Non-Hispanic White

© 2022 Khatana SAM et al. *JAMA Network Open.*

Proportion of residents who are speaks English “Less than Well”
**Housing Type and Transportation Index**
Proportion of housing units that are in multi-unit structures
Proportion of housing units that are mobile homes
Proportion of housing units with more people than rooms
Proportion of households with no vehicle
Proportion of the population living in group quarters

© 2022 Khatana SAM et al. *JAMA Network Open.*

eAppendix 4: Fixed-Effects Model

The fixed effects, or within, estimator is an econometric technique to analyze longitudinal or panel data. This method examines the association between change in the outcome with change in the predictor variable within each subject. The inclusion of subject fixed effects (counties in this analysis) controls for both observed and un-observed time-invariant confounders. The inclusion of time fixed effects accounts for secular time trends that are common for all subjects. The following linear fixed effects model was used:

$$y_{imt} = \beta_1 X_{imt} + a_i + \gamma_m + \zeta_t + \varepsilon_{imt}$$

Where y_{imt} is the mortality for county *i*, in month *m* (May, June, July, August, September), in year *t* (2008 to 2017), X_{imt} is a vector of time-varying independent variables, a_i is the county fixed effect, γ_m is the month fixed effect, ζ_t is the year fixed effect, and ε_{imt} is the error term.

© 2022 Khatana SAM et al. *JAMA Network Open.*

eAppendix 5: Fixed-Effects Model Covariates

The following time-varying, county-level variables were included in the primary fixed effects model:

Monthly – mean precipitation levels, percentage of the population affected by drought, declaration of a disaster by Federal Emergency Management Agency, unemployment rate

Annual – poverty rate, inflation-adjusted median household income, percentage of county residents other than non-Hispanic White, percentage of county-residents aged 65 years and older, percentage of adults with diabetes, percentage of non-elderly adults without health insurance, number of primary care providers and hospital beds per 100,000 individuals

© 2022 Khatana SAM et al. *JAMA Network Open.*

eTable 1: Annual Estimated Additional Deaths Associated With Extreme Heat Days During Summer Months, With Extreme Heat Defined as ≥90°F (32.2°C) and in the 99th Percentile of the Maximum Heat Index in the Baseline Period (1979-2007)

**Year**	**Deaths (95% CI)**
2008	752 (319 to 1,184)
2009	821 (349 to 1,293)
2010	2,284 (971 to 3,597)
2011	2,337 (993 to 3,681)
2012	1,652 (702 to 2,602)
2013	1,060 (451 to 1,670)
2014	977 (415 to 1,539)
2015	1,174 (499 to 1,848)
2016	1,617 (687 to 2,546)
2017	1,060 (451 to 1,670)

© 2022 Khatana SAM et al. *JAMA Network Open.*

eTable 2: Annual Estimated Additional Deaths Associated With Extreme Heat Days During Summer Months, With Extreme Heat Defined as ≥90°F (32.2°C) and in the 95th Percentile of the Maximum Heat Index in the Baseline Period (1979-2007)

**Year**	**Deaths (95% CI)**
2008	964 (519 to 1,409)
2009	1,210 (652 to 1,767)
2010	2,995 (1,613 to 4,377)
2011	2,817 (1,517 to 4,116)
2012	2,150 (1,158 to 3,142)
2013	1,478 (796 to 2,159)
2014	1,327 (715 to 1,939)
2015	1,756 (946 to 2,565)
2016	2,280 (1,228 to 3,332)
2017	1,413 (761 to 2,066)

© 2022 Khatana SAM et al. *JAMA Network Open.*

eTable 3: Annual Estimated Additional Deaths Associated With Extreme Heat Days During Summer Months, With Extreme Heat Defined as ≥90°F (32.2°C) and in the 90th Percentile of the Maximum Heat Index in the Baseline Period (1979-2007)

Year	Deaths (95% CI)
2008	1,089 (523 to 1,654)
2009	1,389 (667 to 2,111)
2010	3,096 (1,487 to 4,704)
2011	2,876.75 (1,382 to 4,372)
2012	2,273.947 (1,092 to 3,456)
2013	1,641.587 (789 to 2,495)
2014	1,493 (717 to 2,269)
2015	1,931 (927 to 2,934)
2016	2,532 (1,216 to 3,847)
2017	1,600 (769 to 2,432)

© 2022 Khatana SAM et al. *JAMA Network Open.*

eReferences:

1. Agency for Toxic Substances and Disease Registry. SVI 2014 Documentation. 2017. https://www.atsdr.cdc.gov/placeandhealth/svi/documentation/pdf/SVI2014Documentation-H.pdf. Published 12/13/2017. Accessed 11/10/2021.
2. Bilal U, Tabb LP, Barber S, Diez Roux AV. Spatial Inequities in COVID-19 Testing, Positivity, Confirmed Cases, and Mortality in 3 U.S. Cities : An Ecological Study. *Ann Intern Med.* 2021.
3. Carmichael H, Moore A, Steward L, Velopulos CG. Using the Social Vulnerability Index to Examine Local Disparities in Emergent and Elective Cholecystectomy. *J Surg Res.* 2019;243:160-164.
4. Neelon B, Mutiso F, Mueller NT, Pearce JL, Benjamin-Neelon SE. Spatial and temporal trends in social vulnerability and COVID-19 incidence and death rates in the United States. *PLoS One.* 2021;16(3):e0248702.

© 2022 Khatana SAM et al. *JAMA Network Open.*
